# nc886 noncoding RNA regulates hepatitis B virus replication via PKR-dependent eIF2α phosphorylation

**DOI:** 10.3389/fcimb.2026.1742078

**Published:** 2026-03-31

**Authors:** Zahra Zahid Piracha, Umar Saeed

**Affiliations:** 1 Szechenyi Istvan University, Egyetem Square 1, Gyor, Hungary; 2 University College, Korea University, Seoul, Republic of Korea; 3 Institute of Graduate Studies and Research, Cyprus International University, Nicosia, Cyprus

**Keywords:** eIF2α phosphorylation, hepatitis B virus (HBV), Huh7 hepatoma cells, integrated stress response, nc886, noncoding RNA, protein kinase R (PKR), translational regulation

## Abstract

**Objective(s):**

Hepatitis B virus (HBV) replication is tightly controlled by host stress and innate immune pathways. The small noncoding RNA nc886 (vtRNA2-1) is a known endogenous inhibitor of protein kinase R (PKR), but its role in HBV biology remains unclear. This study aimed to define the function of the nc886–PKR–eIF2α axis in HBV-replicating hepatoma cells and to determine whether nc886 depletion suppresses HBV replication via PKR-dependent translational control.

**Materials and methods:**

Huh7 cells and Huh7 cells stably harboring a 1.3-mer HBV replicon were used. Endogenous nc886 and PKR expression was assessed by RT-qPCR and Western blot. Loss-of-function experiments employed two independent siRNAs against nc886 and one siRNA against PKR, alone or in combination, with scramble siRNA as control. PKR activation was induced by Poly(I:C); PKR and integrated stress response (ISR) were pharmacologically modulated using C16 (PKR inhibitor) and ISRIB (eIF2B activator), at non-toxic doses defined by MTT assay. Intracellular HBV DNA was measured by Southern blot, HBV pgRNA and subgenomic RNAs by Northern blot and RT-qPCR, and secreted HBsAg/HBeAg by ELISA. PKR–eIF2α–ATF4 signaling was evaluated by Western blot.

**Results:**

nc886 and PKR were efficiently and specifically knocked down without affecting cell viability. nc886 silencing in Huh7–HBV cells increased PKR-dependent eIF2α phosphorylation and ATF4, reduced HBV pgRNA and subgenomic RNAs, and decreased intracellular HBV DNA and secreted HBsAg/HBeAg. PKR knockdown alone slightly enhanced HBV readouts and completely rescued nc886-mediated inhibition of HBV replication and ISR activation in dual-knockdown cells. C16 or ISRIB restored HBV DNA, RNA and antigen production in nc886-silenced or Poly(I:C)-treated cells, while having no effect in control cells, indicating that rescue depended on ISR modulation.

**Conclusion:**

nc886 acts as a critical negative regulator of PKR-dependent ISR signaling during HBV replication in Huh7 cells. Its depletion activates PKR and eIF2α, imposing a translational block that suppresses HBV gene expression. The nc886–PKR–eIF2α module represents a novel host regulatory axis with potential relevance for host-directed HBV therapies.

## Introduction

Chronic hepatitis B virus (HBV) infection remains a major global health problem, affecting nearly 300 million people worldwide and predisposing patients to cirrhosis, liver failure, and hepatocellular carcinoma (GBD 2019 Hepatitis B Collaborators, 2022; World Health Organization, 2024). Despite the availability of effective vaccines and nucleos(t)ide analogues, current therapies rarely eradicate covalently closed circular DNA (cccDNA), the episomal viral minichromosome that persists in hepatocyte nuclei and serves as the template for all viral transcripts (Schreiner and Nassal, 2017; Revill et al., 2019). As a result, HBV can re-activate when treatment is withdrawn or host immunity wanes. There is therefore a pressing need to identify host regulatory pathways that support or restrict HBV replication and could be targeted to complement existing antiviral strategies (Gish et al., 2025).

Innate antiviral signaling is a critical determinant of HBV persistence (Cheng et al., 2017). Pattern recognition receptors detect viral nucleic acids and engage downstream kinases to inhibit translation and promote antiviral gene expression (Hennessy and McKernan, 2021).

Among these pathways, the PKR–eIF2α checkpoint is a central node linking nucleic-acid sensing to rapid translational control. Protein kinase R (PKR), a double-stranded RNA-activated serine/threonine kinase encoded by EIF2AK2, is one of the best-characterized sensors linking RNA recognition to translational control. Upon activation, PKR phosphorylates the α-subunit of eukaryotic initiation factor 2 (eIF2α) at Ser51, thereby blocking the recycling of eIF2-GDP to eIF2-GTP and globally attenuating cap-dependent translation (Gordiyenko et al., 2019; Chung et al., 2025).

This translational shutoff program is commonly referred to as the integrated stress response (ISR). This integrated stress response (ISR) (also referred to as the eIF2α-dependent ISR) limits viral protein synthesis but also induces selected stress-responsive transcripts such as ATF4 (Wek, 2018). Several DNA and RNA viruses have evolved mechanisms to evade or subvert PKR signaling, underscoring its importance in antiviral defense (Beachboard and Horner, 2016). However, the precise contribution of PKR–eIF2α signaling to HBV replication remains incompletely defined, and context-dependent effects have been reported in different experimental systems.

In addition to protein factors, small noncoding RNAs have emerged as key regulators of innate immunity and stress signaling (Liu et al., 2025). The vault RNA vtRNA2-1, also known as nc886, is a 101-nt Pol III–transcribed noncoding RNA that directly binds PKR and maintains it in an inactive state under basal conditions (Lee et al., 2015; Jang et al., 2024). Loss of nc886, either by epigenetic silencing or RNA interference, releases PKR from inhibition, leading to spontaneous PKR activation, eIF2α phosphorylation, and ISR induction (Jeon et al., 2012).

Nc886 therefore represents an upstream “brake” on PKR activation under basal conditions.Nc886 expression is frequently downregulated in malignancies through promoter hypermethylation and has been implicated in cell survival, apoptosis, and oncogenic transformation (Jeon et al., 2012; Lee et al., 2016; Jang et al., 2024). Recent studies further suggest that nc886 might modulate antiviral responses against some RNA viruses, but its role in DNA virus infections, and particularly in HBV replication, has not been systematically explored. Notably, compared with RNA viruses, the contribution of nc886 to host responses during DNA virus infections has received limited attention, and its functional role in HBV replication has not been defined (Li et al., 2015).

Hepatoma cell lines carrying integrated or episomal HBV genomes provide a useful platform to dissect host–virus interactions at the level of cccDNA transcription, RNA processing, and viral protein synthesis (Sells et al., 1987; Ladner et al., 1997). Within this context, the nc886–PKR axis is an attractive candidate regulator of HBV replication because it sits at the interface of RNA sensing and translational control. If nc886 restrains PKR activity in hepatocytes, high nc886 expression would be expected to maintain low basal PKR signaling and permit efficient HBV translation, whereas nc886 depletion should activate PKR, phosphorylate eIF2α, and suppress viral protein synthesis. However, it is not known whether such a mechanism operates in HBV-replicating hepatoma cells, whether the antiviral effect of nc886 loss is fully dependent on PKR, or whether pharmacologic manipulation of the ISR can modulate HBV replication downstream of nc886.

Based on these considerations, we hypothesized that nc886 functions as a negative regulator of PKR-dependent ISR signaling in HBV-replicating Huh7 cells, and that its depletion would activate PKR, increase eIF2α phosphorylation, and consequently inhibit HBV replication at the translational level. To test this hypothesis, we first established a baseline experimental framework by confirming stable endogenous expression of nc886 and PKR in Huh7 cells, validating the efficiency and specificity of nc886 and PKR knockdown, and defining non-toxic concentration ranges for the PKR inhibitor C16 and the ISR modulator ISRIB. We then examined the impact of nc886 silencing on intracellular HBV DNA, HBV RNA transcripts (including pregenomic RNA [pgRNA] and subgenomic RNAs), and secreted HBsAg/HBeAg (hepatitis B surface antigen [HBsAg] and hepatitis B e antigen [HBeAg]), together with PKR–eIF2α activation status, in a Huh7 cell line harboring a 1.3-mer HBV replicon. To determine whether PKR is the critical downstream mediator of nc886, we employed a genetic epistasis strategy combining nc886 and PKR knockdown and assessed rescue of HBV replication and ISR markers. Finally, we used pharmacologic modulation with C16, ISRIB, and the PKR agonist Poly(I:C) (polyinosinic:polycytidylic acid) to interrogate the nc886–PKR–eIF2α axis at different signaling nodes.

By integrating genetic and pharmacologic approaches, this study aims to clarify the functional role of nc886 in HBV replication and to define the extent to which its effects are mediated through PKR-dependent eIF2α phosphorylation. A detailed understanding of this nc886–PKR regulatory module in hepatoma cells may provide mechanistic insight into HBV–host interactions and establish a rationale for future validation in more physiologically relevant models. While therapeutic implications are beyond the scope of this *in vitro* study, these findings may help prioritize ISR-linked host pathways for subsequent translational investigation.

## Materials and methods

### Cell culture, transfection, RNA interference, and molecular analyses

Huh7 human hepatoma cells were maintained in Dulbecco’s modified Eagle’s medium (DMEM; high glucose) supplemented with 10 % heat-inactivated fetal bovine serum (FBS), 100 U/mL penicillin, and 100 µg/mL streptomycin at 37 °C in a humidified 5 % CO_2_ incubator. For HBV replication experiments, we used a Huh7 cell line stably harboring a 1.3-mer HBV genotype D replicon (1.3-mer HBV). Because HBV genotypes can differ in regulatory elements and replication kinetics, findings obtained with a genotype D replicon should be interpreted within this genetic context and may require validation in additional HBV genotypes. Cells were cultured in complete medium containing 400 µg/mL G418 for maintenance and were switched to antibiotic-free medium at least 48 h before experiments. Cells were routinely passaged at 70–80 % confluence and were tested regularly to exclude mycoplasma contamination (Piracha et al., 2020).

### Oligonucleotides and RNA interference

Two independent small interfering RNAs targeting nc886 (si-nc886<ns/>1 and si-nc886<ns/>2) and one siRNA targeting PKR (si-PKR) were designed against human vtRNA2-1/nc886 and EIF2AK2, respectively. A non-targeting siRNA (scramble, Scr) with no homology to human or HBV sequences was used as negative control. All siRNAs were synthesized with standard 21-mer duplex formats and were reconstituted in RNase-free water to 20 µM stocks.

For knockdown experiments, Huh7 or Huh7-HBV-1.3 cells were seeded one day before transfection at 2.0–2.5 × 10^5^ cells/well in 6-well plates or 6–8 × 10³ cells/well in 96-well plates. Transfections were performed in Opti-MEM using Lipofectamine RNAiMAX (Thermo Fisher) according to the manufacturer’s protocol. Unless otherwise indicated, cells were transfected with 50 nM siRNA (Scr, si-nc886#1, si-nc886#2, si-PKR, or the indicated combinations). After 6 h, the transfection mixture was replaced with complete medium. Cells were harvested 24 h or 48 h post-transfection for RNA, protein, viral DNA, antigen, or viability assays as specified.

For experiments requiring dual knockdown (Dual-KD), si-nc886<ns/>1 (25 nM) and si-PKR (25 nM) were co-transfected to maintain a total siRNA concentration of 50 nM, equal to single-siRNA conditions.

### Pharmacologic compounds and treatments

The PKR inhibitor C16 (also known as PKR-Inhibitor, Calbiochem) was dissolved in DMSO to a 10 mM stock and stored at −20 °C. The integrated stress response inhibitor ISRIB was dissolved in DMSO (10 mM stock). High-molecular-weight Poly(I:C) (polyinosinic-polycytidylic acid, a PKR agonist) was reconstituted in sterile water to 1 mg/mL and stored in aliquots at −80°C.

For dose-finding and viability assays, Huh7 cells were exposed to a range of concentrations [C16: 0.1–2.0 µM; ISRIB: 0.02–0.2 µM] for 48 h. For functional rescue experiments, non-toxic working concentrations were used: C16 at 1.0 µM and ISRIB at 0.1 µM unless otherwise stated. Poly(I:C) was applied at 1 µg/mL to activate PKR. Poly(I:C) is a dsRNA mimic that can engage multiple innate sensors (e.g., TLR3 and RLR pathways) in addition to PKR; therefore, in this study Poly(I:C) was used as a general dsRNA/innate activation control to confirm pathway responsiveness rather than as a PKR-specific agonist. DMSO (≤0.1 %, v/v) served as vehicle control in all experiments.

For experiments combining siRNA and drugs, cells were first transfected with siRNAs for 24 h, followed by addition of C16, ISRIB, Poly(I:C), or combinations thereof for an additional 24 h (total 48 h post-transfection at harvest).

### RNA isolation and RT-qPCR

Total RNA was extracted using TRIzol reagent following the manufacturer’s protocol. RNA purity and concentration were measured by spectrophotometry (A_260_/A_280_). For nc886 and PKR mRNA quantification, 1 µg of total RNA was reverse-transcribed using random hexamers and M-MLV reverse transcriptase in a 20 µL reaction.

For nc886, cDNA was generated using a stem-loop primer or gene-specific reverse primer optimized for vtRNA2–1 detection; expression was normalized to U6 snRNA. For PKR mRNA, gene-specific primers were used and expression was normalized to GAPDH. Quantitative PCR was performed using SYBR Green master mix on a real-time PCR system (ABI or equivalent) with the following cycling conditions: 95 °C for 10 min, followed by 40 cycles of 95 °C for 15 s and 60 °C for 60 s. Melting-curve analysis confirmed single-product amplification.

Relative expression levels were calculated using the 2^ΔΔCt^ method with Scr-transfected cells set to 1.0. For HBV RNA quantification accompanying Northern blots, cDNA was synthesized from DNase-treated RNA and amplified with primers specific for HBV core/preC regions; expression was normalized to 18S rRNA.

### Protein extraction and Western blotting

Cells were lysed in ice-cold RIPA buffer (50 mM Tris-HCl, pH 7.4; 150 mM NaCl; 1 % NP-40; 0.5 % sodium deoxycholate; 0.1 % SDS) supplemented with protease and phosphatase inhibitor cocktails. Lysates were incubated on ice for 30 min and clarified by centrifugation at 14 000 ×g for 15 min at 4 °C. Protein concentration was determined using the BCA assay (Saeed and Piracha, 2023).

Equal amounts of protein (30 µg) were mixed with 4× Laemmli buffer, boiled for 5 min, separated by SDS-PAGE on 10–12 % gels, and transferred to PVDF membranes. Membranes were blocked for 1 h at room temperature in 5 % non-fat milk (for total proteins) or 5 % BSA (for phospho-proteins) in TBS-T (Tris-buffered saline, 0.1 % Tween-20), followed by overnight incubation at 4 °C with primary antibodies against PKR, total eIF2α, phospho-eIF2α (Ser51), ATF4, or β-actin (loading control). After washing, membranes were incubated with HRP-conjugated secondary antibodies for 1 h at room temperature.

Signals were visualized using enhanced chemiluminescence (ECL) and captured on X-ray film or a digital imaging system. Band intensities were quantified by densitometry using ImageJ. Phospho-protein levels were normalized to total protein levels, and target proteins were normalized to β-actin.

### HBV DNA extraction and Southern blotting

HBV replicative DNA intermediates were analyzed by Southern blotting using HBV DNA extracted from isolated core particles. Briefly, Huh7-HBV-1.3 cells were harvested and lysed under conditions that preserve nucleocapsids, and core particles were isolated as described previously (Saeed et al., 2021). Encapsidated HBV DNA was released from core particles by proteinase K digestion, extracted, and separated on agarose gels prior to transfer onto nylon membranes. Membranes were UV-crosslinked and hybridized with a DIG-labeled random-primed probe specific for the full-length HBV sequence (derived from the HBV 1.3-mer genotype D construct used in this study). After stringent washing, membranes were incubated with anti-DIG–HRP and developed by chemiluminescence. Replicative intermediates were visualized as relaxed circular (RC) and double-stranded linear (DL) species; where single-stranded (SS) DNA was not consistently prominent under the selected extraction/detection conditions, quantification was performed on the total HBV DNA signal across independent experiments. Band intensities were quantified by densitometry using ImageJ and expressed relative to Scr controls.

### HBV RNA analysis by Northern blotting

Total RNA (20 µg) from Huh7-HBV-1.3 cells was extracted using TRIzol reagent and denatured at 65 °C for 10 min. RNA was separated on 1.2% agarose–formaldehyde gels prepared in 1× MOPS buffer (200 mM MOPS, 10 mM EDTA, 50 mM sodium acetate, pH 7.0) and transferred to nylon membranes followed by UV crosslinking. Membranes were hybridized at 68 °C with a DIG-labeled random-primed probe generated from the full-length HBV sequence (derived from the HBV 1.3-mer genotype D construct used in this study). Signals were visualized by chemiluminescence. The probe detects pgRNA (~3.5 kb) and subgenomic RNAs; the ~2.4 kb and ~2.1 kb subgenomic RNAs may partially comigrate and appear as a single band depending on gel resolution and exposure. Relative HBV RNA levels were quantified by densitometry and normalized to the loading control.

### Measurement of secreted HBsAg and HBeAg

Culture supernatants collected at 48 h post-transfection and/or drug treatment were clarified by centrifugation (1 000 ×g, 5 min) and stored at −20 °C until analysis. Levels of HBsAg and HBeAg were quantified using commercial sandwich ELISA kits according to the manufacturers’ instructions. Absorbance was measured at 450 nm using a microplate reader. To correct for potential differences in cell number or viability, ELISA values were normalized to total cellular protein content or to parallel MTT readings from the same wells and expressed as relative fold change compared with Scr-treated controls.

### Cell viability assay (MTT)

Cytotoxicity of C16 and ISRIB and potential off-target effects of siRNAs were evaluated using the MTT assay. Huh7 cells were seeded in 96-well plates (6–8 × 10³ cells/well) and treated as described. At the indicated time points (24 h and 48 h), 10 µL of MTT solution (5 mg/mL in PBS) was added to each well and incubated for 3–4 h at 37 °C. The medium was carefully removed and formazan crystals were dissolved in 100 µL DMSO. Absorbance at 570 nm (reference 630 nm) was recorded. Cell viability was expressed as percentage of untreated or Scr-transfected control cells.

### Experimental design for epistasis and pharmacologic rescue

For genetic epistasis experiments, Huh7-HBV-1.3 cells were assigned to four groups: Scr, si-nc886, si-PKR, and Dual-KD (si-nc886+si-PKR). All groups were harvested 48 h after transfection for Southern blot, Northern blot/RT-qPCR, ELISA, and Western blot analyses. For pharmacologic modulation experiments, Scr- or si-nc886-transfected cells were treated with C16, ISRIB, Poly(I:C), or combinations as follows: Scr, si-nc886, si-nc886+C16, si-nc886+ISRIB, Poly(I:C), Poly(I:C)+C16, and drug-alone controls (C16, ISRIB). After 48 h, intracellular HBV DNA and RNA, extracellular HBsAg/HBeAg, and p-eIF2α levels were assessed as described above.

Huh7 and Huh7 hepatoma cells were maintained in DMEM, high glucose (Gibco Cat. 11965092) supplemented with 10% heat-inactivated fetal bovine serum, plus penicillin–streptomycin (10,000 U/mL; Cat. 15140122) at 37 °C/5% CO_2_; stable HBV replicon cells were maintained under Geneticin/G418 selection (50 mg/mL; Cat. 10131027) and placed in antibiotic-free medium ≥48 h before experiments. Cells were transfected in Opti-MEM reduced-serum medium (Cat. 31985070) using Thermo Fisher Scientific/Invitrogen Lipofectamine RNAiMAX (Cat. 13778075) with 50 nM total small RNA (single KD: 50 nM; dual KD: 25 nM+25 nM). nc886-targeting “siRNA” sequence is 5′-GGGTCGGAGTTAGCTCAAGCGG-3′, and a widely validated alternative knockdown reagent is the backbone-modified anti-nc886 oligo “anti886 75-56” 5′-mUmCmGmAmACCCCAGCACAmGmAmGmAmU-3′ with matched control “anti-vt 21-2” 5′-mCmCmGmCmUGAGCTAAAGCmCmAmGmCmC-3′. PKR knockdown was performed using a validated EIF2AK2 siRNA mixture with targeting sequences of [5′-UUUACUUCACGCUCCGCCUUCUCGU-3′; 5′-AUGUCAGGAAGGUCA AAUCUGGGUG-3′; and 5′-UUAAGUUCCUCCAUGAAGAAACCUG-3′]. Total RNA was isolated with TRIzol Cat. 15596026 and, where specific enzymes were used, first-strand cDNA synthesis was performed with M-MLV reverse transcriptase Cat. 28025013. qPCR primer sequences include nc886-F 5′-CGGGTCGGAGTTAGCTCAAGCGG-3′ and nc886-R 5′-AAGGGTCAGTAAGCACCCGCG-3′; 18S-F 5′-CGGCTTTGGTGACTCTAGAT-3′ and 18S-R 5′-GCGACTACCATCGAAAGTTG-3′; GAPDH-F 5′-CATCAAGAAGGTGGTGAAGCAGG-3′ and GAPDH-R 5′-AGTGGTCGTTGAGGGCAATGC-3′; and U6-F 5′-CTCGCTTCGGCAGCACA-3′ with U6-R 5′-AACGCTTCACGAATTTGCGT-3′. Encapsidated HBV DNA replicative intermediates were analyzed by Southern blot and HBV RNAs by Northern blot using DIG-labeled random-primed HBV probes prepared/detected with a DIG-High Prime DNA Labeling and Detection kit (Roche DIG-High Prime kit SKU 11745832910). Cells were lysed in RIPA buffer, total protein was quantified by BCA (Pierce BCA kit Cat. 23227), and immunoblots were probed using PKR (D7F7) rabbit mAb Cat. 12297 (1:1000), phospho-eIF2α (Ser51) (119A11) rabbit mAb Cat. 3597, total eIF2α (D7D3) rabbit mAb Cat. 5324 (1:1000), ATF4 (D4B8) rabbit mAb Cat. 11815 (1:1000), and β-actin (8H10D10) mouse mAb Cat. 3700. C16 (PKR inhibitor; CAS 608512-97-6; MedChemExpress HY-10343) and ISRIB (eIF2B activator; CAS 1597403-47-8; Sigma-Aldrich SML0843) were prepared as 10 mM stocks in DMSO and used at working concentrations C16 1.0 μM; ISRIB 0.1 μM, while high-molecular-weight Poly(I:C) was prepared at 1 mg/mL and used at 1 μg/mL. Secreted HBsAg were quantified by HBsAg ELISA kit Abbexa abx351801 and HBeAg were quantified by HBeAg ELIS kit Cell Biolabs QuickTiter VPK-5003-5. The Cell viability was assessed by MTT Cat. M5655.

### Densitometry and statistical analysis

ImageJ software was used for densitometric analysis of Southern, Northern, and Western blots. For each experiment, background-subtracted band intensities were normalized to appropriate loading controls (β-actin for proteins) and expressed as fold change relative to Scr controls.

All experiments were performed in at least three independent biological replicates with technical duplicates or triplicates where applicable. Data are presented as mean ± standard deviation (SD). Statistical analyses were conducted using GraphPad Prism. Comparisons between two groups were performed using unpaired two-tailed Student’s t-tests, while multiple group comparisons were analyzed by one-way ANOVA followed by Tukey’s *post-hoc* test. A p-value < 0.05 was considered statistically significant, with *p < 0.05, **p < 0.01, and ***p < 0.001 used in figures and legends. Non-significant differences are indicated as “ns”. For blot-based densitometry (Western, Southern, Northern), quantification was performed from at least three independent biological experiments unless otherwise stated in the figure legend. For each independent experiment, densitometry was calculated from the corresponding blot and then averaged across experiments to generate mean ± SD.

## Results

### Baseline expression and tool validation in Huh7 cells

**Figure 1:**
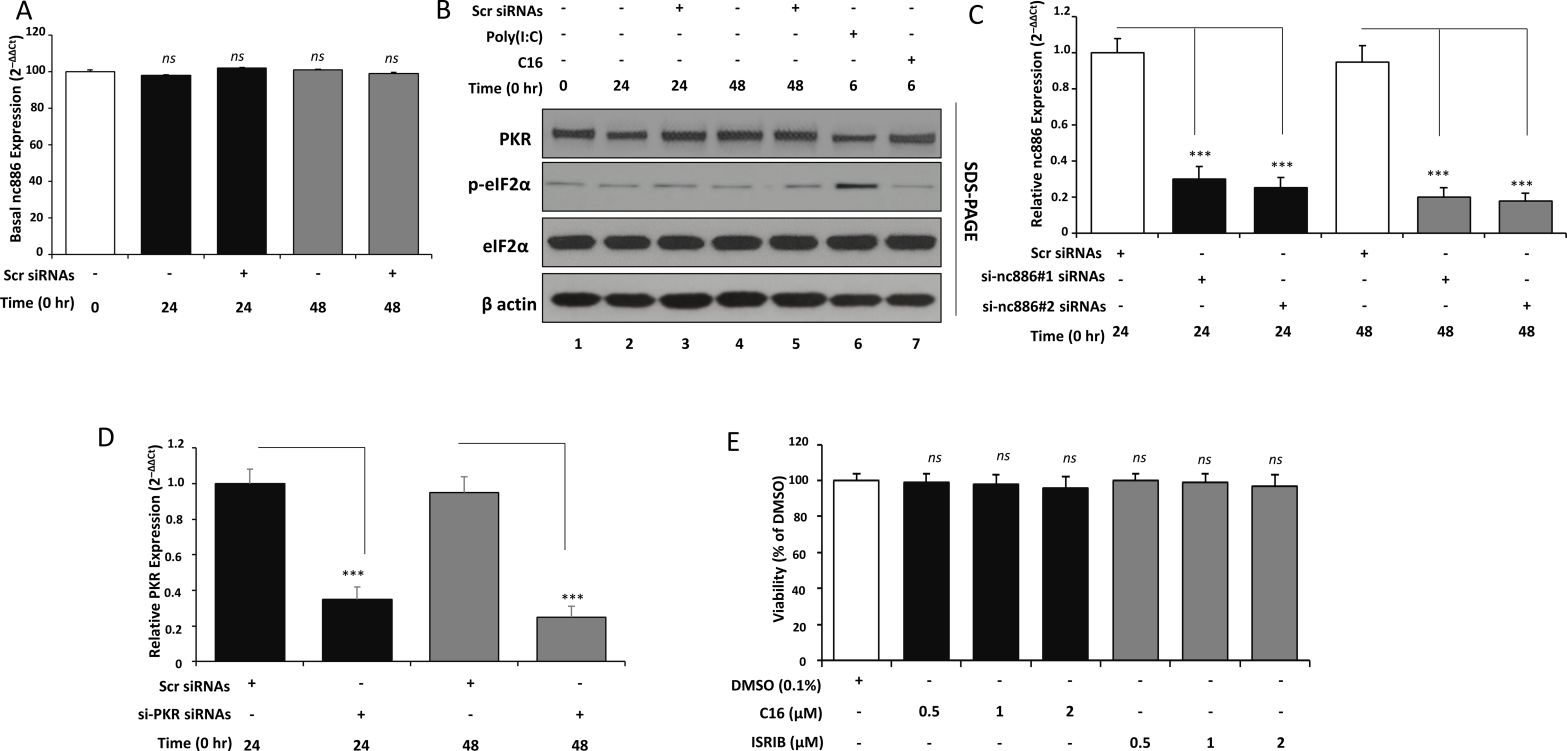
Baseline expression and validation of genetic and pharmacologic tools in Huh7 cells. **(A)** Basal nc886 expression in Huh7 cells evaluated by RT-qPCR. Cells were mock-transfected or transfected with non-targeting scramble siRNA (Scr; 50 nM) using Lipofectamine RNAiMAX and harvested at 24 h and 48 (h) Total RNA was isolated with TRIzol, reverse-transcribed using random primers, and nc886 levels were quantified by SYBR Green RT-qPCR and normalized to U6 snRNA using the 2^-^ΔΔCt method. Data are expressed as fold change relative to mock (set to 1.0).**(B)** Baseline PKR–eIF2α signaling and responsiveness to pharmacologic modulation. Huh7 cells were left untreated (basal), treated with Poly(I:C) (1 µg/mL, 6–8 h) to activate PKR, or pretreated with C16 (1 µM, 1 h) followed by Poly(I:C). Whole-cell lysates were analyzed by Western blot using antibodies against total PKR, phospho-eIF2α (Ser51), total eIF2α, and β-actin. Equal protein loading (20–30 µg per lane) was confirmed by β-actin. Representative blots and densitometric quantification of p-eIF2α normalized to total eIF2α are shown. Densitometry was performed from n = 3 independent biological experiments. **(C)** Validation of nc886 knockdown. Huh7 cells were transfected with Scr, si-nc886<ns/>1, or si-nc886<ns/>2 (50 nM) and harvested at 24 h and 48 (h) Total RNA was extracted and nc886 expression was quantified by RT-qPCR as in **(A)**. Bars represent relative nc886 levels normalized to U6 and expressed as fold change versus Scr.**(D)** Validation of PKR knockdown. Huh7 cells were transfected with Scr or si-PKR (50 nM) and harvested at 24 h and 48 (h) PKR mRNA was quantified by RT-qPCR, normalized to GAPDH, and expressed relative to Scr. In parallel, PKR protein levels were assessed by Western blot using anti-PKR and β-actin antibodies to confirm knockdown at the protein level. **(E)** Determination of non-toxic working concentrations of C16 and ISRIB by MTT assay. Huh7 cells were treated for 48 h with increasing concentrations of C16 (0.1–2.0 µM) or ISRIB (0.02–0.2 µM). Cell viability was assessed by incubation with MTT (5 mg/mL, 3–4 h), dissolution of formazan crystals in DMSO, and measurement of absorbance at 570 nm. Viability is expressed as percentage of untreated control. Data in all panels represent mean ± SD of at least three independent experiments performed in duplicate or triplicate. Statistical analysis was performed by one-way ANOVA with Tukey’s *post hoc* test; ns, not significant; ***p < 0.001 versus Scr or indicated control. Densitometry was performed from n = 3 independent biological experiments. Bars represent mean ± SD of densitometry values from n = 3 independent biological experiments.

The primary objective of this experiment was to establish a validated baseline framework for nc886–PKR pathway studies in Huh7 cells by confirming endogenous expression stability, verifying knockdown efficiency, and defining non-toxic concentrations for pharmacological modulators. Quantitative RT-PCR analysis normalized to U6 revealed that nc886 expression remained unchanged in both mock-transfected and scramble (Scr) control cells at 24 and 48 hours (*ns*, *p* > 0.05), with fold changes near unity. These findings confirm that transfection reagents or scrambled siRNA sequences do not alter basal nc886 transcriptional levels (**Figure 1A**).

Western blot analysis demonstrated the baseline activity and responsiveness of the PKR–eIF2α axis under defined conditions (**Figure 1B**). Huh7 cells exhibited low constitutive PKR and phosphorylated eIF2α (p-eIF2α) levels under basal conditions, whereas exposure to the PKR activator Poly(I:C) markedly enhanced p-eIF2α. Pretreatment with the PKR inhibitor C16 strongly reduced p-eIF2α without altering total PKR or eIF2α levels, validating the functional specificity and sensitivity of the PKR–eIF2α assay system.

Two independent nc886-targeting siRNAs (si-nc886<ns/>1 and si-nc886<ns/>2) significantly suppressed nc886 expression by approximately 70–80 % at both 24 and 48 hours relative to Scr controls (*p* < 0.001), confirming robust and sequence-specific gene silencing (**Figure 1C**). Similarly, RT-qPCR quantification demonstrated efficient PKR knockdown, with PKR mRNA reduced to approximately 35 % after 24 hours and 25 % after 48 hours compared with Scr controls (*p* < 0.001) (**Figure 1D**).

Cell-viability profiling indicated that the PKR inhibitor C16 and the integrated-stress-response inhibitor ISRIB were well tolerated within defined dose ranges. Huh7 cells maintained ≥ 93–97 % viability following 48-hour exposure to C16 (≤ 1 µM) and ISRIB (≤ 0.2 µM), with only a mild, non-significant reduction (~ 87 %) observed at 2 µM C16 (*ns*, *p* > 0.05) (**Figure 1E**). These results established non-toxic working concentrations for subsequent functional assays.

Our data demonstrates that nc886 and PKR can be selectively and efficiently silenced, that the PKR–eIF2α pathway provides a sensitive and quantifiable readout of pathway modulation, and that pharmacological tools such as C16 and ISRIB can be applied safely within non-toxic ranges to interrogate nc886–PKR signaling dynamics in downstream experiments.

### nc886 knockdown suppresses HBV replication via PKR activation and eIF2α phosphorylation

To determine whether nc886 influences HBV replication, Huh7 cells stably harboring the 1.3-mer HBV replicon were transfected with either scramble (Scr) or nc886-targeting siRNAs (#1 and #2) and analyzed 48 hours post-transfection, corresponding to maximal nc886 depletion (**Figure 1C**).

**Figure 2:**
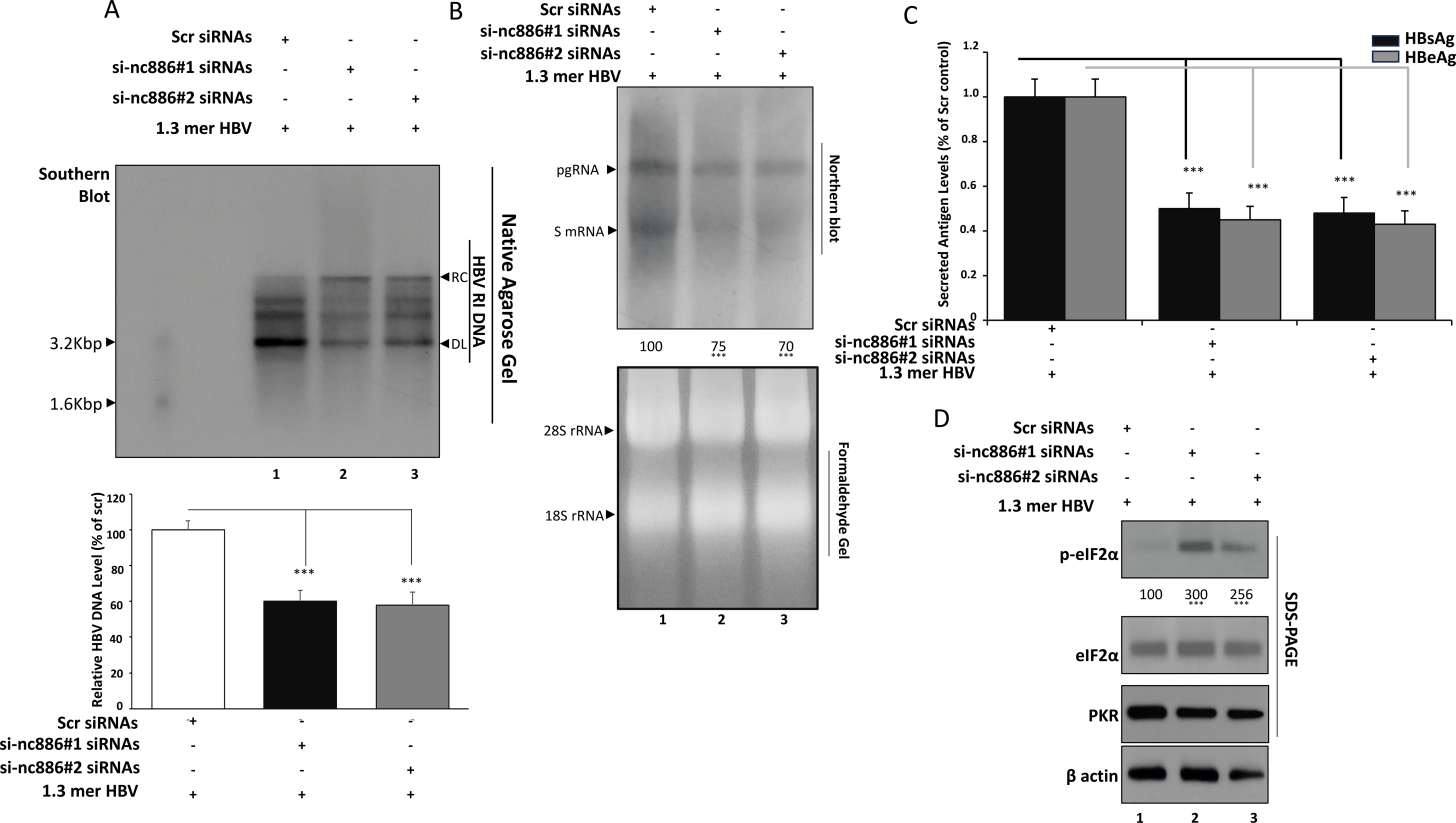
nc886 knockdown modulates HBV replication and PKR–eIF2α signaling in Huh7–HBV 1.3-mer cells. **(A)** Analysis of intracellular HBV DNA by Southern blot. Huh7 cells stably harboring a 1.3-mer HBV replicon were transfected with Scr, si-nc886<ns/>1, or si-nc886<ns/>2 (50 nM) and harvested at 48 (h) HBV DNA was extracted from isolated core particles (encapsidated replicative intermediates), released by proteinase K digestion, extracted by phenol–chloroform, and separated on agarose gels. DNA was transferred to nylon membranes and hybridized with a DIG-labeled random-primed probe specific for the full-length HBV sequence (derived from the HBV 1.3-mer genotype D construct used in this study). Relaxed circular (RC) and double-stranded linear (DL) HBV DNA species were visualized by chemiluminescence. Where single-stranded (SS) DNA was not consistently prominent under these conditions, quantification was performed using the total HBV DNA signal. Densitometric quantification of total HBV DNA is shown as relative signal normalized to Scr. **(B)** HBV RNA analysis by Northern blot and RT-qPCR. Total RNA was extracted from the same cultures and resolved on formaldehyde–agarose gels, transferred to nylon membranes, and probed with a DIG-labeled random-primed probe specific for the full-length HBV sequence (derived from the HBV 1.3-mer genotype D construct used in this study) to detect pregenomic RNA (pgRNA, ~3.5 kb) and subgenomic RNAs (sRNAs, ~2.4/2.1 kb). Under these gel conditions, the ~2.4 kb and ~2.1 kb transcripts may partially comigrate and can appear as a single subgenomic band. Densitometric analysis of pgRNA and sRNAs normalized to loading control is shown. Inset/companion bar graphs, where applicable, depict HBV RNA levels quantified by RT-qPCR and normalized to 18S rRNA. **(C)** Quantification of secreted HBsAg and HBeAg by ELISA. Culture supernatants collected at 48 h post-transfection were clarified and analyzed for HBsAg and HBeAg using commercial ELISA kits according to the manufacturers’ protocols. Absorbance at 450 nm was measured, and antigen levels were normalized to total cellular protein or parallel MTT readings from the corresponding wells. Data are expressed as relative antigen levels compared with Scr-treated cells. **(D)** Western blot analysis of PKR–eIF2α signaling after nc886 knockdown. Whole-cell lysates from Scr- and si-nc886–transfected Huh7–HBV 1.3-mer cells were subjected to SDS-PAGE and immunoblotting for total PKR, phospho-eIF2α (Ser51), total eIF2α, and β-actin. Representative blots and densitometric ratios of p-eIF2α to total eIF2α are presented. Data represent mean ± SD from at least three independent experiments. Statistical significance was calculated using one-way ANOVA with appropriate *post hoc* tests; ns, not significant; ***p < 0.001 versus Scr. Bars represent mean ± SD of densitometry values from n = 3 independent biological experiments.

Southern blot analysis revealed a moderate but reproducible reduction in intracellular HBV DNA forms following nc886 silencing (**Figure 2A**). Quantification indicated that total HBV DNA (relaxed circular and double-stranded linear species) declined by approximately 40 % compared with Scr controls (*p* < 0.001). These results suggest that nc886 depletion partially impairs the synthesis or accumulation of HBV replicative intermediates.

Northern blotting using an HBV core/preS probe showed clear reductions in HBV pregenomic RNA (pgRNA; ~3.5 kb) and subgenomic RNAs (sRNAs; ~2.4/2.1 kb) (**Figure 2B**). Densitometric analysis indicated that pgRNA decreased to about 70–75 % of Scr levels, supporting a partial transcriptional and/or stability effect at the RNA level.

Consistent with these findings, ELISA-based quantification revealed that secreted HBsAg and HBeAg were significantly decreased in nc886-silenced cells (**Figure 2C**). Both viral antigens dropped to approximately half of Scr control levels (*p* < 0.001), indicating that nc886 depletion reduces viral protein production and secretion.

Western blot analysis demonstrated that nc886 knockdown markedly increased phosphorylated eIF2α (Ser51) levels without affecting total PKR or total eIF2α abundance (**Figure 2D**). Densitometric analysis revealed a ~3-fold rise in the p-eIF2α relative to Scr controls, consistent with PKR activation. β-actin served as the loading control.

Our data suggest that nc886 silencing activates the PKR–eIF2α pathway, leading to translational inhibition that reduces HBV RNA, DNA, and antigen levels. The data support a model in which nc886 normally restrains PKR activity, and its depletion triggers an antiviral stress response that suppresses HBV replication through impaired RNA translation and viral protein synthesis.

### Genetic epistasis confirms PKR as the downstream effector of nc886

**Figure 3:**
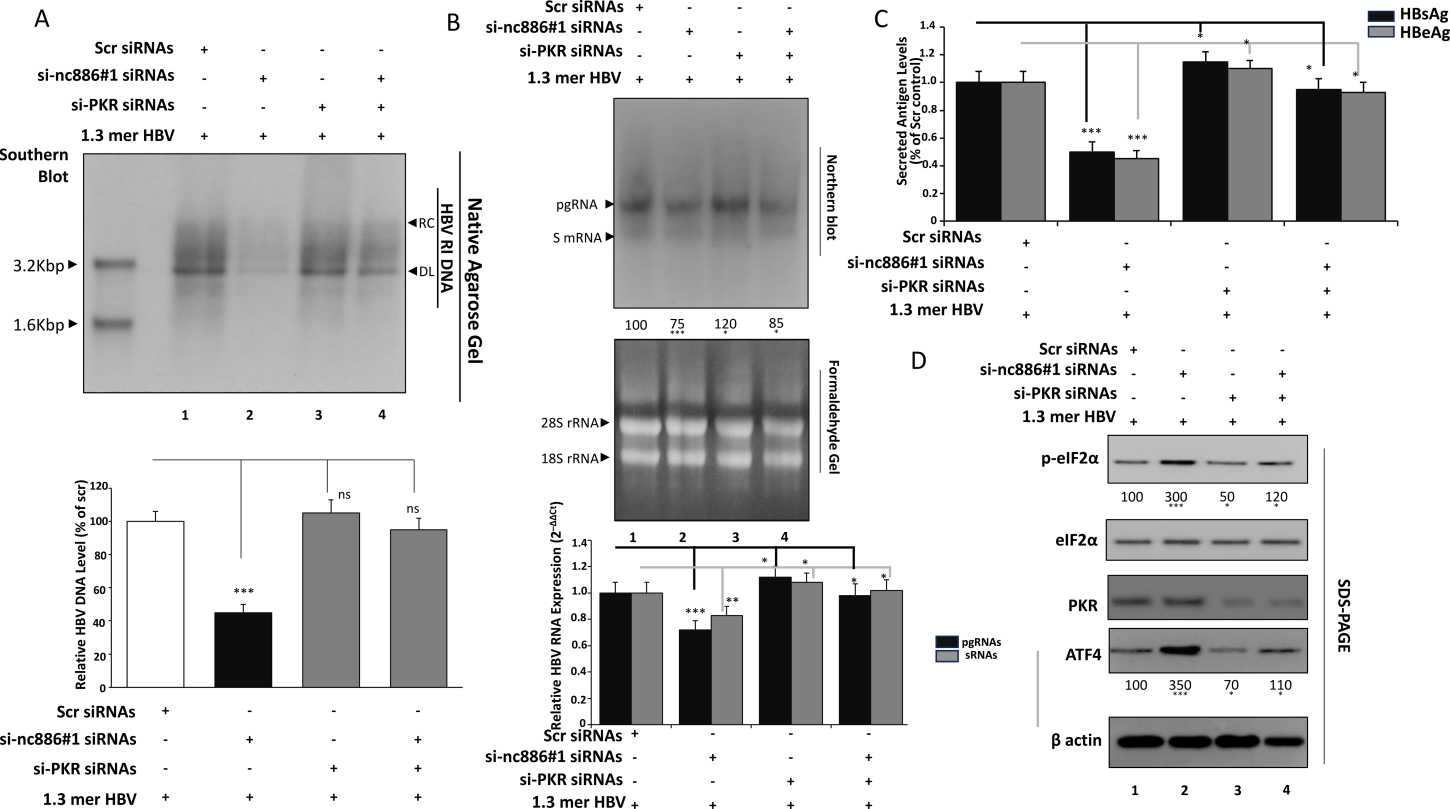
Genetic epistasis between nc886 and PKR in regulating HBV replication and integrated stress response. **(A)** Southern blot analysis of HBV DNA in single and dual knockdown conditions. Huh7–HBV 1.3-mer cells were transfected with Scr, si-nc886, si-PKR, or both si-nc886 and si-PKR (Dual-KD), keeping total siRNA at 50 nM. Cells were harvested 48 h post-transfection. HBV DNA was extracted from isolated core particles (encapsidated replicative intermediates) and analyzed by Southern blot as described in **Figure 2A**. RC and DL DNA forms were visualized, and where SS DNA was not consistently prominent under these conditions, total HBV DNA signal was quantified by densitometry and expressed relative to Scr. **(B)** HBV RNA expression under epistasis conditions. Total RNA was extracted from the same cultures and resolved on formaldehyde–agarose gels, transferred to nylon membranes, and probed with a DIG-labeled random-primed probe specific for the full-length HBV sequence (derived from the HBV 1.3-mer genotype D construct used in this study) to detect pregenomic RNA (pgRNA, ~3.5 kb) and subgenomic RNAs (sRNAs, ~2.4/2.1 kb). Under these gel conditions, the ~2.4 kb and ~2.1 kb transcripts may partially comigrate and can appear as a single subgenomic band. Membranes were reprobed for 18S rRNA or GAPDH as loading controls. Densitometric analysis of pgRNA and sRNAs normalized to loading control is shown. In the lower panel, RT-qPCR was performed on DNase-treated RNA using HBV core-specific primers, with normalization to 18S rRNA. Data are expressed as fold change relative to Scr. **(C)** Secreted HBsAg and HBeAg in nc886 and PKR single/dual knockdown cells. Culture supernatants from Scr, si-nc886, si-PKR, and Dual-KD groups were collected at 48 h and analyzed for HBsAg and HBeAg by ELISA. Antigen levels were normalized to total cellular protein or viability (MTT) and expressed as relative values compared with Scr. **(D)** Western blot assessment of PKR–eIF2α–ATF4 signaling. Whole-cell lysates from the four groups were analyzed by SDS-PAGE and immunoblotting for total PKR, phospho-eIF2α (Ser51), total eIF2α, ATF4, and β-actin. Representative blots are shown together with densitometric ratios (p-eIF2α/total eIF2α and ATF4/β-actin) normalized to Scr. Data represent mean ± SD of at least three independent experiments. Statistical comparisons were performed using one-way ANOVA with Tukey’s *post hoc* test; ns, not significant; *p < 0.05; **p < 0.01; ***p < 0.001 versus Scr or indicated groups. Bars represent mean ± SD of densitometry values from n = 3 independent biological experiments.

To confirm whether PKR mediates the antiviral effects induced by nc886 depletion, Huh7 cells stably expressing the HBV 1.3-mer replicon were transfected with scramble control (Scr), si-nc886, si-PKR, or both (Dual-KD). All analyses were conducted 48 hours post-transfection, corresponding to the validated time point of maximal gene knockdown. Southern blotting (**Figure 3A**) demonstrated that nc886 silencing sharply reduced all HBV DNA forms, while PKR depletion alone slightly enhanced replication although non-significant. Densitometric quantification showed that total HBV DNA declined to approximately 45 % of Scr after nc886 knockdown, increased modestly to about 105 % with si-PKR (*ns*), and was restored to around 95 % under Dual-KD conditions. This data clearly demonstrates that co-depletion of PKR rescued HBV DNA copy numbers close to baseline levels. These data establish that the replication defect induced by nc886 loss is mediated through PKR activation.

Next, Northern blotting (**Figure 3B**, upper panel) and RT-qPCR (**Figure 3B**, lower panel) revealed a moderate reduction in RNAs 25% upon nc886 knockdown. Conversely, PKR silencing alone led to a mild elevation (120%). Dual-KD restored both RNA species to approximately 1.0-fold relative to Scr. This reciprocal response demonstrates that removal of PKR alleviates nc886-induced transcriptional repression, consistent with secondary transcriptional stabilization following relief of translational stress.

Furthermore, ELISA analyses of extracellular viral antigens reflected these replication trends (**Figure 3C**). nc886 knockdown markedly reduced secreted HBsAg (0.50 ± 0.07) and HBeAg (0.45 ± 0.06) compared with Scr, while PKR depletion slightly enhanced antigen levels (HBsAg 1.15 ± 0.07; HBeAg 1.10 ± 0.06). Dual-KD restored antigen secretion to near-control levels (HBsAg 0.95 ± 0.08; HBeAg 0.93 ± 0.07), confirming that PKR activity governs the nc886-dependent suppression of viral protein synthesis and release.

Western blot analysis (**Figure 3D**) demonstrated that total PKR and total eIF2α were unchanged in si-nc886 cells but PKR reduced in si-PKR and Dual-KD groups. In contrast, phospho-eIF2α (Ser51) increased approximately three-fold and ATF4 by about 3.5-fold upon nc886 knockdown, indicating activation of the integrated stress response (ISR). Both p-eIF2α and ATF4 signals returned to baseline upon co-depletion of PKR, confirming that nc886 loss triggers ISR activation in a PKR-dependent manner.

Collectively, the genetic epistasis experiments establish PKR as the essential downstream effector of nc886. Loss of nc886 activates PKR-dependent phosphorylation of eIF2α, leading to translational repression, ATF4 induction, and reduced HBV RNA, DNA, and antigen output. Co-silencing PKR abrogates these effects, demonstrating that the antiviral outcome of nc886 depletion is entirely mediated through the PKR–eIF2α signaling axis.

### Pharmacologic modulation reinforces the nc886–PKR axis in HBV replication

To further substantiate the functional linkage between nc886 suppression and PKR activation, pharmacologic modulation experiments were performed in Huh7 cells harboring the HBV 1.3-mer replicon. C16 (a selective PKR inhibitor) and ISRIB (an integrated stress response inhibitor acting at eIF2B) were used to evaluate rescue potential, while Poly(I:C) served as a PKR-activating mimic. All treatments were applied at non-toxic doses determined in **Figure 1E**, with harvest at 48 h.

**Figure 4:**
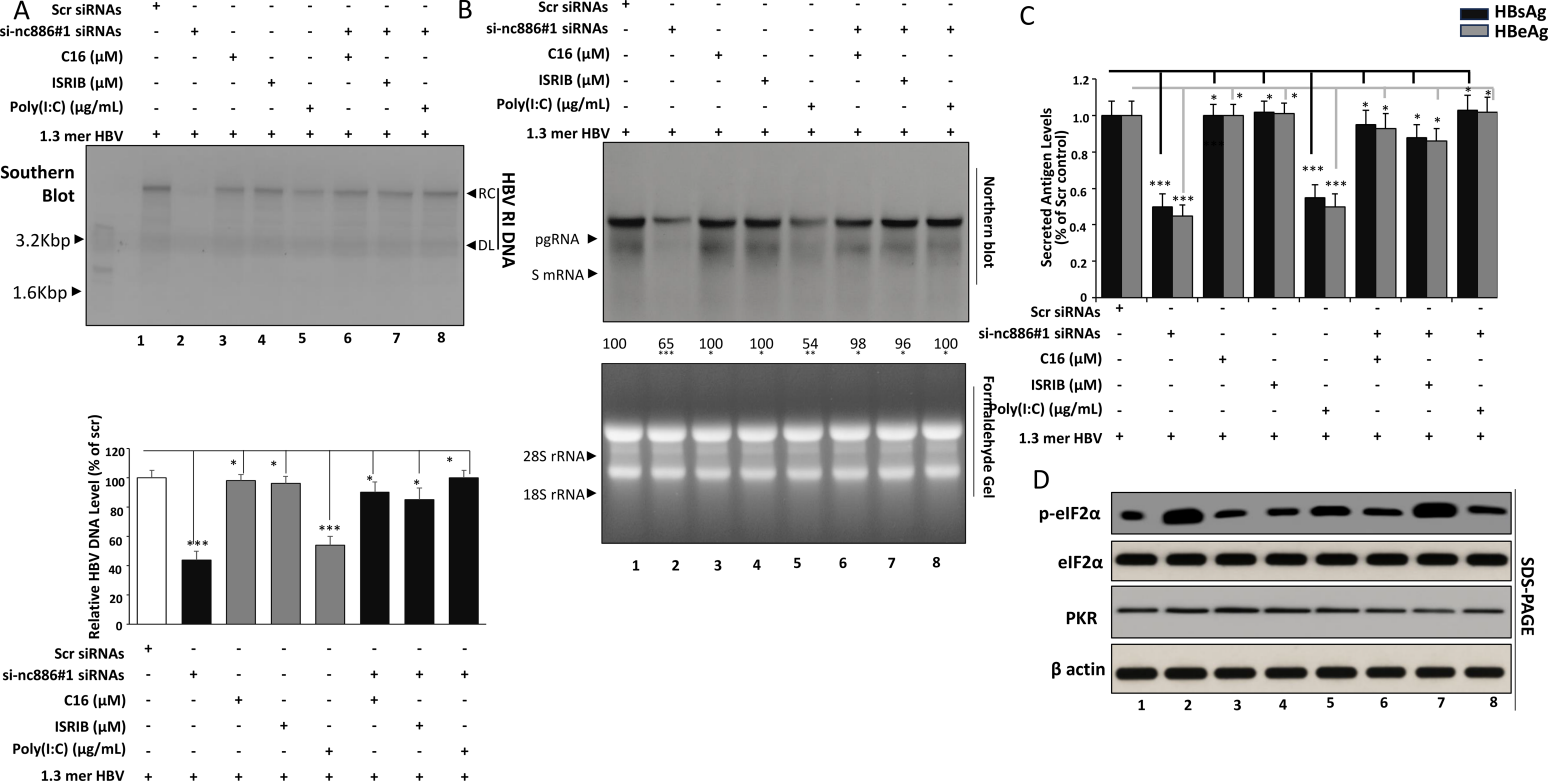
Pharmacologic modulation of PKR–eIF2α signaling confirms nc886–PKR axis in HBV-replicating Huh7 cells. **(A)** Effect of PKR inhibition and ISR bypass on HBV DNA under nc886 knockdown or Poly(I:C) treatment. Huh7–HBV 1.3-mer cells were transfected with Scr or si-nc886 (50 nM) and, after 24 h, treated with C16 (1 µM), ISRIB (0.1 µM), Poly(I:C) (1 µg/mL), or their combinations (si-nc886+C16, si-nc886+ISRIB, Poly(I:C)+C16). DMSO served as vehicle control. At 48 h, nuclear DNA was extracted and analyzed by Southern blot as described in **Figure 2A**. RC, DL and SS HBV DNA forms were detected, and total HBV DNA was quantified by densitometry and expressed relative to Scr+DMSO. **(B)** HBV RNA expression under pharmacologic modulation. Total RNA was extracted from the same cultures and resolved on formaldehyde–agarose gels, transferred to nylon membranes, and probed with a DIG-labeled random-primed probe specific for the full-length HBV sequence (derived from the HBV 1.3-mer genotype D construct used in this study) to detect pregenomic RNA (pgRNA, ~3.5 kb) and subgenomic RNAs (sRNAs, ~2.4/2.1 kb). Under these gel conditions, the ~2.4 kb and ~2.1 kb transcripts may partially comigrate and can appear as a single subgenomic band. Densitometric analysis of pgRNA and sRNAs normalized to loading control is shown. Densitometric analysis of pgRNA and sRNA bands normalized to the loading control is shown. Where indicated, complementary RT-qPCR was performed on HBV transcripts with normalization to 18S rRNA. **(C)** Secreted HBsAg and HBeAg in response to nc886 knockdown and PKR/ISR modulation. Culture supernatants were collected at 48 h and analyzed for HBsAg and HBeAg by ELISA. Values were normalized to total protein or viability and expressed as relative levels compared with Scr+DMSO. Drug-alone groups (C16, ISRIB) and Poly(I:C) controls were included to assess baseline effects of pharmacologic agents. **(D)** Western blot analysis of p-eIF2α in pharmacologically modulated conditions. Whole-cell lysates from Scr, si-nc886, Poly(I:C), and their combinations with C16 or ISRIB were analyzed by Western blot using antibodies against total PKR, phospho-eIF2α (Ser51), total eIF2α, and β-actin. Representative blots and densitometric quantification of p-eIF2α/total eIF2α ratios are shown. Data represent mean ± SD from at least three independent experiments. Statistical analysis was performed by one-way ANOVA with appropriate *post hoc* tests; ns, not significant; *p < 0.05; **p < 0.01; ***p < 0.001 versus Scr+DMSO or as indicated. Bars represent mean ± SD of densitometry values from n = 3 independent biological experiments.

Southern blot analysis (**Figure 4A**) revealed that si-nc886 markedly suppressed HBV DNA accumulation, reducing relaxed circular (RC), and single-stranded (SS) forms to ~44 % of control. PKR inhibition by C16 restored replication to ~90 %, while ISRIB yielded a similar rescue (~85 %), confirming that both upstream and downstream ISR blockade reversed the inhibitory effect of nc886 loss. Poly(I:C) phenocopied nc886 depletion, lowering HBV DNA to ~54 % of control, whereas co-treatment with C16 restored it to ~100 %. Neither C16 nor ISRIB alone altered HBV replication, validating their neutrality at the chosen concentrations.

Northern blotting (**Figure 4B**) demonstrated that si-nc886 and Poly(I:C) each reduced pgRNA and sRNA expression to ~70–80 % of baseline. These decreases were fully normalized by either C16 co-treatment or ISRIB co-treatment, whereas Poly(I:C)+C16 restored transcripts to >100 % of control. Drug-alone treatments showed no change. The consistency between RNA and DNA restoration supports that nc886 regulates HBV post-transcriptionally via the PKR–eIF2α pathway.

ELISA measurements of HBsAg and HBeAg paralleled nucleic acid findings (**Figure 4C**). si-nc886 and Poly(I:C) each decreased antigen release by ~50 %, whereas addition of C16 or ISRIB rescued secretion to near-control levels. Dual treatments (Poly(I:C)+C16 or si-nc886+ISRIB) normalized HBsAg/HBeAg secretion (0.88–1.03-fold vs Scr), while C16-only and ISRIB-only groups remained indistinguishable from control, demonstrating that rescue effects were context-dependent. Western blotting revealed that total PKR and total eIF2α were unchanged across all groups, but phospho-eIF2α (Ser51) increased nearly three-fold after nc886 knockdown or Poly(I:C) exposure, confirming ISR activation (**Figure 4D**). C16 co-treatment sharply reduced p-eIF2α (~120 % of control), whereas ISRIB did not alter phosphorylation yet fully restored HBV output, indicating a translational bypass downstream of p-eIF2α. β-Actin remained constant, validating equal loading.

Collectively, these pharmacologic data reaffirm that nc886 suppresses HBV replication through PKR-dependent eIF2α phosphorylation, resulting in a translational block rather than transcriptional repression. C16 and ISRIB each restore viral replication by acting at distinct nodes of the ISR cascade, C16 upstream at PKR, ISRIB downstream at eIF2B, thereby confirming the mechanistic specificity and robustness of the nc886–PKR regulatory axis in HBV biology.

## Discussion

In this study, we identify the noncoding RNA nc886 as a critical negative regulator of PKR-dependent integrated stress signaling during HBV replication in Huh7 cells. Using combined genetic and pharmacologic approaches, we show that nc886 knockdown activates PKR and eIF2α phosphorylation, leading to reduced intracellular HBV DNA, diminished pgRNA and subgenomic RNA levels, and decreased secretion of HBsAg and HBeAg. Co-silencing PKR or pharmacologic inhibition of PKR with C16, as well as translational bypass of the integrated stress response with ISRIB, restored viral replication and normalized ISR markers. These findings support a model in which nc886 restrains PKR activity under basal conditions, whereas loss of nc886 unleashes PKR–eIF2α signaling and imposes a translational block on HBV gene expression.

Our data extend the emerging concept that nc886/vtRNA2–1 acts as an endogenous rheostat of PKR (Jeon et al., 2012; Lee et al., 2016). Biochemical studies demonstrated that nc886 directly binds PKR and prevents its dimerization and autophosphorylation, thereby maintaining low basal PKR activity in resting cells (Calderon and Conn, 2017). Subsequent work in cancer models showed that epigenetic silencing of nc886 leads to constitutive PKR activation, eIF2α phosphorylation, and ATF4 induction, with context-dependent consequences ranging from apoptosis to growth arrest or adaptation to stress (Lee et al., 2014). We recapitulate this core signaling module in a virological setting: nc886 knockdown in HBV-replicating Huh7 cells produced robust induction of p-eIF2α and ATF4 without altering total PKR or eIF2α levels, and these ISR changes were completely reversed by PKR co-silencing. Thus, the nc886–PKR axis is functional in hepatoma cells and can be engaged to modulate viral replication.

**Figure 5:**
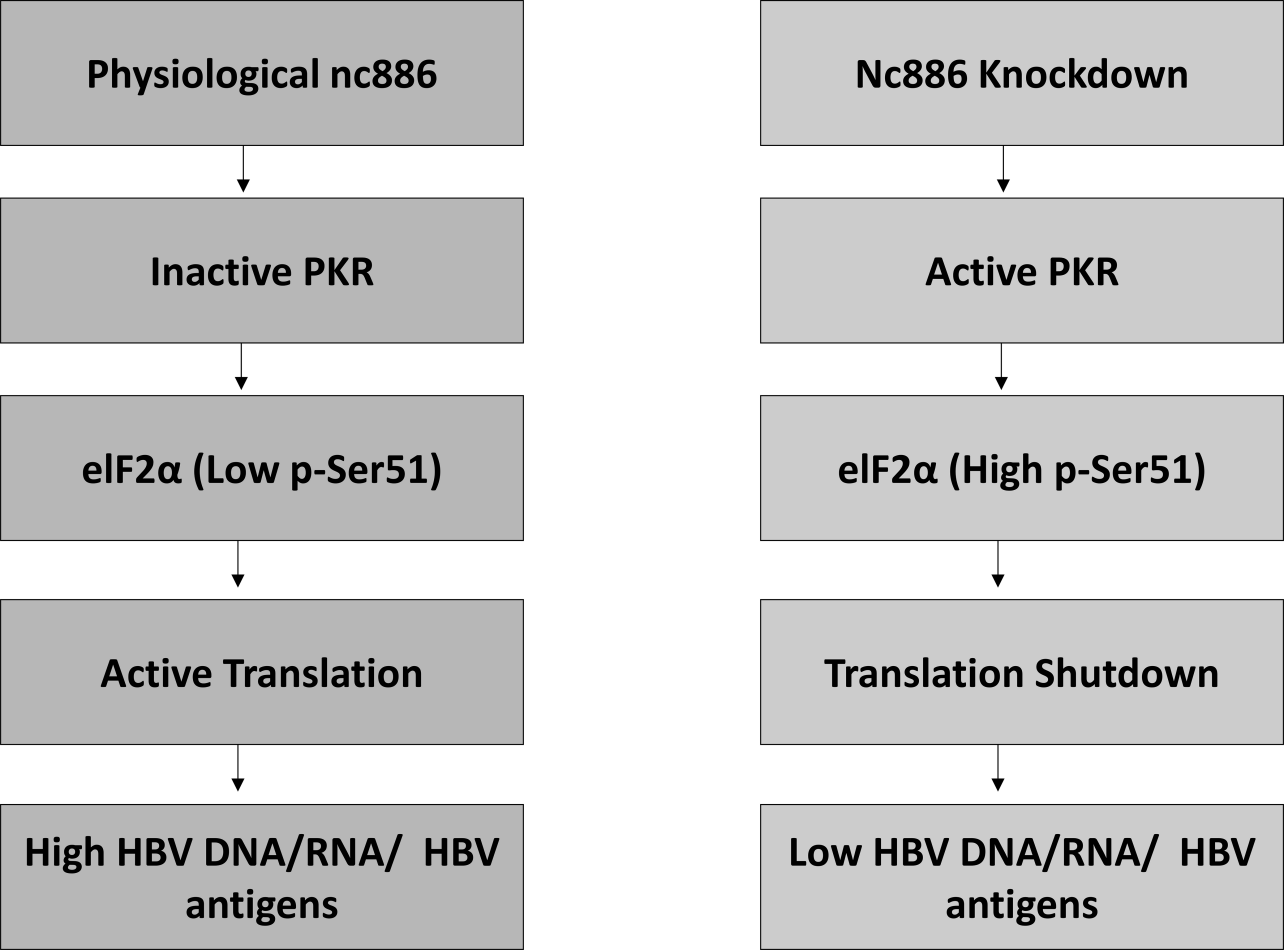
Proposed model of nc886–PKR–eIF2α signaling and its impact on HBV replication. **Figure 5**. Schematic representation of the regulatory role of nc886 in PKR-dependent integrated stress signaling during HBV replication. Left panel: Under physiological conditions, nc886 (vtRNA2-1) binds and restrains PKR, maintaining PKR in an inactive state. Consequently, eIF2α phosphorylation at Ser51 remains low, allowing efficient cap-dependent translation and supporting normal synthesis of HBV proteins, accumulation of viral RNA and DNA, and secretion of viral antigens.Right panel: Loss or knockdown of nc886 releases PKR from repression, resulting in PKR activation and increased phosphorylation of eIF2α at Ser51. This induces a translational shutdown, reducing production of core and polymerase proteins required for pgRNA encapsidation and reverse transcription. As a result, intracellular HBV DNA and RNA decline, and secretion of HBsAg and HBeAg is diminished.This model integrates the genetic (si-nc886, si-PKR) and pharmacological (C16, ISRIB) rescue experiments described in the study, demonstrating that nc886 regulates HBV replication primarily through PKR-mediated control of translation rather than direct transcriptional effects.

As illustrated in **Figure 5**, under physiological conditions, nc886 maintains PKR in an inactive state, resulting in low phosphorylation of eIF2α (Ser51) and efficient cap-dependent translation, which supports normal production of HBV RNAs, viral proteins, and replicative intermediates. In contrast, nc886 knockdown releases PKR from inhibition, leading to PKR activation and elevated p-eIF2α, which imposes a global translational shutdown. This translational block reduces synthesis of core and polymerase proteins, thereby limiting pgRNA encapsidation and reverse transcription, ultimately resulting in reduced intracellular HBV DNA, lower viral RNA abundance, and decreased secretion of HBsAg and HBeAg. This mechanistic model reconciles our genetic (si-nc886 and si-PKR) and pharmacologic (C16 and ISRIB) rescue experiments, demonstrating that nc886 modulates HBV replication primarily through PKR-dependent control of translation rather than direct transcriptional regulation.

The role of PKR in HBV infection has remained controversial. Early reports indicated that HBV polymerase and HBx can interfere with PKR activation or its interaction with eIF2α, thereby protecting viral translation (Zhao et al., 2021), whereas other studies observed that PKR activation inhibits HBV replication by blocking pgRNA translation and encapsidation (Park et al., 2011). Differences in model systems (transient transfection versus stable replicons or infection models), viral genotype, and the presence or absence of interferons or synthetic dsRNA agonists have contributed to these discrepancies (Ladner et al., 1997). In our stable 1.3-mer replicon model, basal PKR and p-eIF2α levels were low, and specific activation of PKR by Poly(I:C) or nc886 depletion consistently suppressed HBV DNA, RNA, and antigen production, while genetic or pharmacologic inhibition of PKR restored viral outputs. These observations argue that, at least in Huh7 replicon cells, PKR functions as a bona fide antiviral effector against HBV, in line with studies showing that PKR overexpression or exogenous activation represses HBV translation (Han et al., 2011). Importantly, we used C16 at concentrations that reduced p-eIF2α without affecting total PKR, and ISRIB rescued HBV replication without changing phosphorylation status, supporting the conclusion that the critical step is eIF2α-dependent translational control rather than non-specific toxicity.

Our work also contributes to the broader literature on noncoding RNAs as modulators of HBV–host interactions. Multiple microRNAs, including miR-122, miR-125a-5p and miR-199a-3p, have been reported to regulate HBV replication by targeting viral transcripts or host factors involved in cccDNA transcription and stability (Chen et al., 2011; Mirzaei et al., 2023). Long noncoding RNAs induced by HBx or inflammatory signaling similarly influence HBV gene expression and hepatocarcinogenesis (Qiu et al., 2017). In contrast, nc886 is a Pol III–transcribed small ncRNA primarily known as a PKR regulator rather than a canonical miRNA or lncRNA (Rajić et al., 2025). Our findings show that nc886 indirectly controls HBV replication by tuning a central stress-kinase pathway, adding a new layer to ncRNA-mediated control in HBV biology. Given that nc886 is frequently hypermethylated and silenced in hepatocellular carcinoma (Lee, 2023), it is tempting to speculate that reduced nc886 in tumor tissue might favor chronic PKR activation and ISR adaptation, with complex effects on both viral persistence and tumor progression.

Mechanistically, our data indicate that nc886 knockdown primarily exerts its antiviral effect at the level of translation, with secondary consequences for viral RNA and DNA accumulation. We observed a modest reduction (~25–30 %) in pgRNA and subgenomic RNAs by Northern blot, contrasted with a more pronounced decrease in viral DNA and secreted antigens (~50–60 %). This apparent discrepancy is consistent with a primarily translational restriction: eIF2α phosphorylation can rapidly suppress cap-dependent translation, thereby reducing viral protein output even when RNA levels decline only modestly over the same time window. Because HBV DNA synthesis depends critically on the availability of core and polymerase proteins for pgRNA encapsidation and reverse transcription, relatively small reductions in RNA, or even unchanged RNA with reduced translation, can produce larger downstream decreases in replicative DNA intermediates and secreted antigens. In addition, HBV RNAs may exhibit comparatively slow turnover in hepatoma systems, creating a temporal lag in RNA loss relative to protein and DNA readouts, while stress responses such as stress-granule formation and ISR-linked RNA handling could further influence RNA stability in a time-dependent manner. Co-depletion of PKR normalized p-eIF2α and ATF4 and restored viral DNA, RNA, and antigens to near-control levels, and ISRIB rescued HBV replication without altering p-eIF2α abundance. These patterns are most parsimoniously explained by a translational block imposed by PKR-dependent eIF2α phosphorylation, which reduces synthesis of core and polymerase proteins necessary for reverse transcription and virion production and may secondarily influence cccDNA transcription or pgRNA stability through feedback mechanisms (Zheng and Bevilacqua, 2004). We did not directly quantify cccDNA copy number or chromatin marks, so subtle transcriptional effects cannot be excluded; however, the dominance of translational rescue by ISRIB argues that the nc886–PKR axis acts mainly at the level of protein synthesis rather than cccDNA epigenetics.

The combined genetic and pharmacologic approaches used here provide internal validation for the specificity of the nc886–PKR–eIF2α pathway. Single-gene knockdowns can be confounded by off-target effects, particularly for small noncoding RNAs (Kim et al., 2024). The use of two independent siRNAs against nc886 that produced comparable phenotypes, together with rescue by PKR co-silencing and two mechanistically distinct ISR modulators (C16 upstream at PKR, ISRIB downstream at eIF2B), makes an off-target explanation unlikely. Moreover, neither C16 nor ISRIB alone altered HBV replication at the non-toxic doses selected, indicating that they do not directly enhance or suppress HBV in the absence of stress signaling. Instead, their ability to normalize HBV replication only when PKR is activated (by nc886 knockdown or Poly(I:C)) supports a context-dependent effect that is tightly linked to ISR status.

From a translational perspective, our findings raise the possibility that deliberate engagement of the nc886–PKR axis could be harnessed to suppress HBV replication. In principle, strategies that mimic nc886 loss, such as antisense oligonucleotides or small molecules disrupting nc886–PKR binding, might activate PKR and impose a reversible translational brake on HBV, complementing nucleos(t)ide analogues that target polymerase and reduce viremia (Lee et al., 2019). However, chronic PKR activation is associated with apoptosis, inflammation, and metabolic dysregulation in the liver (Gal-Ben-Ari et al., 2019) thus, systemic manipulation of this pathway carries a substantial risk of toxicity. Conversely, pharmacologic ISR inhibition by ISRIB has shown neuroprotective and metabolic benefits in preclinical models (Rabouw et al., 2019), but our data warn that ISRIB can restore HBV replication in the setting of PKR activation. These considerations underscore the need to carefully balance antiviral efficacy and host-cell homeostasis when targeting ISR components in chronic HBV infection.

Several limitations of this study should be acknowledged. First, all experiments were performed in Huh7 hepatoma cells carrying an integrated 1.3-mer replicon, which does not recapitulate the full cccDNA life cycle, viral entry via NTCP, or the complex cytokine milieu of chronic infection. Future work in NTCP-expressing hepatocyte models, primary human hepatocytes, or humanized-liver mice will be required to confirm whether the nc886–PKR axis operates similarly in more physiological settings. Second, we focused on nc886 knockdown and did not perform complementary overexpression experiments; such gain-of-function studies would help determine whether supraphysiologic nc886 levels can further enhance HBV replication or confer resistance to interferon-induced PKR activation. Third, we used p-eIF2α and ATF4 as surrogate markers of ISR activation but did not conduct unbiased transcriptomic profiling to delineate downstream stress-responsive gene programs that might also contribute to the antiviral state (Sells et al., 1987; Ladner et al., 1997). Next, we did not dissect potential cross-talk between PKR and other HBV-relevant pathways, such as PERK, GCN2, mTOR, or autophagy, which may modify the net effect of ISR modulation on viral replication and cell fate. PERK and GCN2 are additional eIF2α kinases activated by ER stress and amino-acid deprivation, respectively, and could therefore reinforce or substitute for PKR in driving eIF2α phosphorylation and ATF4-linked ISR outputs in hepatocytes. In parallel, mTORC1 regulates cap-dependent translation (via 4E-BP1/S6K) and autophagy, providing a plausible intersection with PKR–eIF2α signaling that may collectively tune HBV protein availability required for pgRNA encapsidation and reverse transcription. A limitation of this study is that all mechanistic and functional experiments were performed using a genotype D 1.3-mer replicon in Huh7 cells; given known genotype-dependent differences in HBV regulatory sequences and replication behavior, it will be important to test whether nc886 depletion elicits comparable PKR–eIF2α/ISR activation and HBV restriction across other genotypes (e.g., A–C and E/F) using parallel replicon and/or infection models. Furthermore, analyses were performed primarily at 48 h post-transfection; future work incorporating earlier (e.g., 12–24 h) and later (e.g., 72 h) time points will be valuable to map the temporal sequence of PKR–eIF2α/ISR activation relative to changes in HBV RNA, DNA intermediates, and antigen secretion.

Environmental and food–water exposure studies collectively highlight that infection risk is strongly shaped by microbial pressures and host stress-response pathways (Zseni et al., 2019; Tema et al., 2020; Ashaolu et al., 2021; Greff et al., 2021; Korcz et al., 2021; Pancza et al., 2021; Greff et al., 2022; Greff et al., 2023; Tihanyi-Kovács et al., 2023; Greff et al., 2026). Building on this context, our work shows that HBV exploits the nc886–PKR–eIF2α stress-signaling axis to regulate viral replication. Despite these limitations, the present work provides a coherent and experimentally supported model in which nc886 functions as a gatekeeper of PKR-dependent ISR signaling in HBV-replicating hepatoma cells. By demonstrating that nc886 depletion activates PKR, induces eIF2α phosphorylation and ATF4, and suppresses HBV replication in a manner that is reversible by PKR knockdown or ISR modulation, we link a specific noncoding RNA to a well-defined antiviral effector pathway. These insights broaden the understanding of how small noncoding RNAs integrate stress and innate immune signals in chronic viral infection and suggest that the nc886–PKR–eIF2α module may represent a targetable node for future host-directed therapies in HBV.

## Data Availability

The original contributions presented in the study are included in the article/supplementary material. Further inquiries can be directed to the corresponding author.
